# Persona-mediated dissociation: a new framework for understanding the work/self-split in sex workers

**DOI:** 10.3389/fpubh.2026.1779090

**Published:** 2026-03-12

**Authors:** Ellis Sather

**Affiliations:** Department of Counseling, Loyola University New Orleans, New Orleans, LA, United States

**Keywords:** persona-mediated dissociation, sex worker mental health, dissociation in sex workers, occupational identity, emotional labor and dissociation, identity compartmentalization, adaptive dissociation

## Abstract

Research on dissociation in sex workers is sparse, dated, and frequently framed through trauma-exclusive or symptom-focused models, leaving the occupational functions of dissociative processes underexamined. In contrast, qualitative and ethnographic scholarship consistently describes intentional work persona construction as routine in sex work and often articulated in dissociative-like terms. This Hypothesis and Theory article proposes *persona-mediated dissociation*, a framework describing context-specific, identity-mediated psychological distancing in which a deliberately constructed work persona mediates between the private self and the interpersonal, emotional, and embodied demands of sexual labor. The model specifies voluntariness, contextual specificity, reversibility, and preserved executive functioning as core dimensions and predicts that persona activation will co-vary with state dissociative phenomenology and maintained functioning. Distress is expected primarily when persona use is prolonged, role exit is impaired, or structural constraints limit decompression and support. This framework clarifies distinctions between occupational regulation and clinically significant dissociation and generates testable predictions for future research.

## 1 Introduction

Dissociation refers to disruptions in the normal integration of consciousness, memory, identity, emotion, perception, body representation, motor control, and behavior (1). Contemporary models increasingly conceptualize dissociation as a spectrum of regulatory responses rather than a unitary marker of psychopathology, with voluntariness, contextual specificity, reversibility, and functional impact distinguishing adaptive psychological distancing from maladaptive or clinically significant dissociation (1–3). Within this framing, dissociative-like experiences may function protectively under high interpersonal or affective demand yet become distressing when responses become rigid, persist beyond the eliciting context, or are accompanied by loss of control (4–6).

Sex work can be understood to include the exchange of sexual services, performances, or erotic labor for material or financial compensation across diverse venues, including street-based work, escorting, brothel-based services, stripping and exotic dance, pornography, camming, phone sex, digital content creation platforms, and other forms of sexual labor (7, 8, 45). Experiences within sex work vary considerably as a function of intersecting structural and individual factors, including work venue, age, race and ethnicity, socioeconomic status, migration status, legal context and criminalization, exposure to violence, stigma and discrimination, disability, and sexual and gender identity (7–10).

In sex work, dissociative phenomena have been documented, but the empirical literature remains limited, dated, and methodologically heterogeneous, with core studies constrained in generalizability (11–15). At the same time, ethnographic and qualitative research consistently describes deliberate work persona construction and identity management as routine occupational practice, often articulated by workers in language resembling dissociative phenomenology while remaining intentional and context-bound (16–21).

To bridge this gap, this paper proposes *persona-mediated dissociation* as a theory-building framework describing context-specific, identity-mediated psychological distancing in which a deliberately constructed work persona mediates between the private self and the interpersonal, emotional, and embodied demands of sexual labor (2, 16, 18, 20, 21). Drawing on dissociation theory, emotional labor research, and performance psychology, the framework centers role transitions and boundary integrity as candidate mechanisms shaping whether persona use functions as adaptive regulation or contributes to distress (5, 15, 22–25).

## 2 Understanding dissociation

Across trauma, cognitive, and neurobehavioral models, key dimensions of voluntariness, context specificity, reversibility, and functional impact are central to distinguishing adaptive dissociative responses from maladaptive or clinically significant dissociation (1–3). When affective arousal is modulated or partially distanced, experiences are more likely to be encoded in verbally accessible, contextualized memory systems rather than as fragmented, sensory-dominant traces associated with intrusive re-experiencing (2, 26). Conversely, overwhelming affect and loss of regulatory control during exposure are associated with fragmented encoding, reduced narrative coherence, and increased risk for persistent dissociative symptoms (27, 28).

Recent empirical syntheses further support this distinction, demonstrating that dissociative experiences are more strongly associated with current affective dysregulation and stress states than with trauma history alone (4, 5). Researchers also reported that the functional meaning and clinical impact of dissociation also vary by developmental timing and context, with dissociation related to adult stressors operating through different mechanisms than dissociation rooted in early trauma (29).

### 2.1 Adaptive, maladaptive, and clinical dissociation

Adaptive dissociation refers to context-bound, flexible regulatory responses that involve partial psychological distancing while preserving overall functioning, agency, and continuity of identity (1–3). Such dissociative states are typically voluntary or semi-volitional, activated in response to specific situational demands, and reversible once precipitating conditions subside (2, 3). Recent empirical syntheses further support this regulatory framing, demonstrating that dissociative experiences are more strongly associated with current affective dysregulation and situational stress states than with trauma history alone, underscoring their state-dependent and potentially adaptive nature (4, 5). Within this framework, dissociation may function as a short-term protective mechanism that allows individuals to modulate affective intensity and maintain task engagement under conditions of heightened interpersonal or emotional demand.

In contrast, maladaptive dissociation is characterized by increasing rigidity, persistence beyond the original context, or diminished voluntary control, such that dissociative responses begin to generate subjective distress or interfere with emotional processing and role functioning (2, 27). Critically, maladaptive dissociative responses still retain some degree of contextual linkage and identity continuity (2, 6). Empirical studies of depersonalization and emotional numbing indicate that dissociative experiences may initially be experienced as protective or functional yet become distressing when emotional blunting persists beyond the context in which it was adaptive (6, 30). Contemporary findings further suggest that dissociation associated with ongoing affective dysregulation or chronic stress states may contribute to impaired psychological flexibility and integration, even in the absence of severe or early trauma exposure (4, 5).

Clinical dissociation, as defined within DSM-5-TR criteria, is distinguished not by the mere presence of dissociative phenomena, but by their involuntary nature, pervasiveness across contexts, impaired reversibility, and association with clinically significant distress or functional impairment, often accompanied by fragmented memory encoding and persistent re-experiencing (1, 26, 27). Trauma-informed clinical models emphasize that dissociation becomes clinically concerning when it shifts from a flexible, context-bound regulatory response to a rigid, intrusive pattern that interferes with identity coherence, emotional processing, interpersonal functioning, or occupational performance (2, 27).

## 3 Brief literature overview—dissociation in sex workers

With respect to dissociation among sex workers, the majority of available empirical studies are more than a decade old, methodologically heterogeneous, and limited in generalizability (11–13, 31). Moreover, these foundational studies reflect the historical and sociopolitical contexts in which they were produced; they often used childhood trauma–oriented frameworks and, at times, adopted moralizing perspectives, while giving comparatively little attention to sex worker agency (12, 13, 31).

Nonetheless, these legacy studies provide descriptively rich accounts documenting diverse manifestations of dissociative features among sex workers. Across studies, dissociation is described as encompassing both clinically distressing symptoms associated with traumatic exposure and context-specific psychological distancing used to manage everyday occupational demands, with adaptive and distressing dissociative processes sometimes co-occurring within the same individuals (11, 14, 20, 21, 31). More recent publications addressing dissociation among sex workers have largely taken the form of reviews or secondary syntheses, frequently reiterating the conceptual framing and pathologizing assumptions of earlier studies rather than advancing new empirical evidence or theoretical development (13, 15, 31).

To address these gaps, reflect personal experiences, and synthesize interdisciplinary data, the present paper proposes the construct of *persona-mediated dissociation*, conceptualizing a pattern of context-specific dissociative processes in which a deliberately constructed work persona mediates between the individual’s private self and the emotional and interpersonal demands of sexual labor, such that the persona engages the interaction while the self-experiences psychological distancing or altered self-experience. In this model, dissociation is structured and contained through the occupational persona rather than occurring as a diffuse loss of integration, and may function adaptively or distressingly depending on context, reversibility, and intentional initiation. This paper aims to: synthesize fragmented interdisciplinary findings; identify gaps in both empirical literature and theory; propose a new framework; advance sex work research; encourage further empirical research; support client-responsive, sex work–informed counseling practices and interventions; and inform more accurate, stigma-responsive public health and mental health services (1, 5, 11, 13, 20, 21).

### 3.1 Patterns in existing research—identity fragmentation

Within a small and methodologically heterogenous base of literature, Tschoeke et al. (13) reiterated a strong prevalence of dissociative phenomena in sex workers including amnesia, depersonalization, derealization, absorption, and dissociative disorders. However, these studies were predominantly symptom-focused and interpreted within a trauma-focused framework emphasizing childhood sexual abuse rather than foregrounding sex workers’ contextualized descriptions of occupational regulation. Researchers also identified substantial gaps in the dissociation literature on sex workers, including minimal assessment of identity-related dissociation.

Notably, the primary studies in the review revealed accounts consistent with identity fragmentation and self-state differentiation; Ross et al. (31) reported that sex workers described clinically maladaptive dissociative phenomenon as well as adopting a different identity state during sex work (13). Further, Cooper et al. (44) indicated that sex workers were significantly more likely to recall work-related experiences from an observer (third-person) perspective, a memory style associated with dissociation, emotional detachment, and reduced subjective distress. Notably, Cooper et al. (11, 44) described sex workers shifting between field (first-person embodied recall) and observer perspectives, rather than exhibiting sustained dissociative pathology.

Although work-related identity dissociation has received little systematic attention in contemporary empirical research or theory-building, identity-based dissociative experiences are documented in the available data (11, 13, 14, 20, 21). These findings suggest that sex workers report a broad range of dissociative symptoms and features, including experiences consistent with identity fragmentation and psychological identity distancing during occupational performance (11, 13, 14, 20, 21).

## 4 Qualitative and ethnographic descriptions of work personas in sex workers

Aligned with this pattern of identity-related dissociation described in empirical research, ethnographic and qualitative studies provide critical insight into how sex workers experience and manage identity processes within occupational contexts (16–21). Across this literature, sex workers are consistently described as engaging in deliberate and self-aware identity management practices that shape how emotional, embodied, and interpersonal labor is performed (17–21).

Sanders (20, 21) documents that sex workers “create a manufactured identity specifically for the workplace as a self-protection mechanism to manage the stresses of selling sex” (p. 322), a process that participants describe as intentional, learned, and context-dependent. Workers repeatedly articulate this identity shift using language that parallels dissociative phenomenology, while situating it explicitly within work-related performance: “You go into a mode… I become her” (p. 329); “She acts differently than I would ever do” (p. 329); and “You kind of believe it yourself, but of course I do this for the money” (p. 334). Importantly, these descriptions emphasize conscious role entry rather than loss of control, with workers expressing awareness of when and why the persona is activated.

Similar patterns are documented across ethnographic studies, which describe paid sexual encounters as involving the deliberate adoption of a different self, often explicitly framed as acting or performance, through which sex workers report being, feeling, and behaving differently from their everyday sense of self (16, 17, 20, 21). Pseudonyms, selectively altered personality traits, and curated or fabricated biographical details are described as core components of this work persona, enabling workers to present a coherent and desirable client-facing identity while maintaining separation from their private selves (16, 19–21).

Recent qualitative work further underscores the intentional and strategic nature of persona construction; Daniel et al. (18) described how sex workers consciously develop and manage distinct professional personas to regulate stigma exposure, preserve privacy, and maintain psychological boundaries across work and non-work contexts. Participants in the study emphasized that personas were actively crafted and monitored, particularly in public-facing environments, reinforcing that identity separation functions as a protective and agentic strategy rather than an unconscious or imposed process (18).

### 4.1 Function of the work persona in sex workers

Across studies, the work persona is documented to serve three interrelated functions: (a) emotional labor oriented toward meeting clients’ erotic expectations, (b) a safety and privacy strategy that reduces risks of identification, stalking, and doxxing, and (c) a psychological boundary that separates paid sexual performance from the worker’s private self (16, 18, 19, 21).

Qualitative analyses suggest that sex workers’ personas mediate client projections, entitlement, and objectification by situating these interactions as occupational rather than self-referential (16, 17, 20, 21). Ethnographic accounts describe the work persona as a boundary-maintenance and interactional power strategy, enabling sex workers to regulate client behavior while outwardly performing compliance, rather than reflecting internalized objectification or diminished agency (16, 20, 21). Feminist scholarship cautions that such strategic compliance should not be interpreted as evidence of internalized objectification, instead emphasizing the importance of contextualized readings of agency, power, and self-protection (46).

Taken together, this body of qualitative and ethnographic research demonstrates that sex workers intentionally construct and inhabit work personas with clear awareness of their functions and boundaries; personas can be understood as not merely defensive but purposeful (16–19, 21).

## 5 Emotional labor theory

Understanding and theorizing how sex workers intentionally construct and sustain work personas necessitates engagement with emotional labor theory which provides a pre-existing framework for examining how internal experience and outward expression are regulated in occupational contexts (16, 17, 20–22, 32). Emotional labor refers to the regulation of emotional expression to meet occupational display demands; the worker remains themself while managing what behavior and emotion is shown. Emotional labor researchers consistently distinguished between surface acting, which involves modifying outward emotional displays without changing internal feelings, and deep acting, which involves actively shaping internal emotions, appraisals, or attentional focus to align felt experience with role expectations (23, 32). Despite the use of the term *acting*, emotional labor theory is conceptually distinct from performance psychology; here, surface and deep acting refers to emotion-regulation processes grounded in emotional labor theory (32), rather than theatrical role immersion, which will be addressed subsequently.

### 5.1 Emotional labor theory—surface vs. deep acting

In emotional labor theory, surface acting (defined as the regulation of outward emotional expression without corresponding changes in internal experience) has been shown to be insufficient for sustaining roles that must be perceived as authentic or emotionally convincing over time (22, 32, 47). Instead, such roles are more effectively sustained through deep acting, which involves regulating internal emotional and cognitive processes to align with role expectations, thereby reducing emotional dissonance relative to surface acting (22, 24, 32, 33, 47).

Contemporary empirical work demonstrates that these strategies have distinct psychological consequences: surface acting is robustly associated with emotional dissonance, exhaustion, burnout, and adverse mental health outcomes, whereas deep acting shows more variable and context-dependent effects (33, 34). A recent meta-analysis found that surface acting and emotional dissonance were reliably linked to depression, anxiety, insomnia, and poorer overall mental health, while deep acting showed no consistent association with negative mental health outcomes at the aggregate level (33). Daily diary and multilevel studies further indicate that surface acting more reliably undermines after-work recovery, whereas deep acting may sometimes support recovery or interpersonal functioning, though these effects are inconsistent and contingent on contextual factors such as perceived gratitude, interaction quality, and regulatory effort (47, 48).

Importantly, contemporary emotional labor research emphasizes that deep acting reflects emotion-regulation processes such as cognitive reappraisal and attentional modulation, which may reduce emotional dissonance when successful, but can still carry regulatory costs when sustained or poorly supported (33, 48).

### 5.2 Emotional labor theory—emotional processing

Beyond organizing behavior and performance, these role-based deep acting experiences also shape how stress and threat are subjectively experienced during high-intensity interpersonal exposure, consequently influencing how occupational demands are appraised, managed, and psychologically encoded within context (22–24).

Emotional-labor research indicates that when role-based emotion regulation is engaged, effective role enactment selectively constrains affective activation by amplifying task-relevant emotions while suppressing vulnerability-associated states (22–24, 32, 33). Emotional labor research suggests that role-based enactment allows workers to experience stressful or challenging interactions through a professional role enactment rather than the private self, which is associated with reduced self-referential appraisal and clearer identity boundaries (22, 23, 32, 35).

#### 5.2.1 Emotional labor theory—psychological strains

Within emotional labor theory, adverse outcomes of sustained emotion regulation are commonly organized around difficulties disengaging from display rules (role exit), the transfer of regulatory strain into non-work domains (spillover), and impaired recovery between regulated and unregulated emotional states (transition difficulties), particularly under conditions of repeated surface acting, emotional dissonance, and prolonged interpersonal demand (22–24). Critically, these adverse effects of poor emotional labor involve depleted self-regulatory resources, leading to exhaustion, not overexposure or boundary failure, which is better addressed in the performance psychology section.

#### 5.2.2 Spillover

Emotional labor theory explains spillover as the transfer of work-related emotion regulation strain into non-work domains due to depletion of self-regulatory resources (23, 48). Surface acting is particularly associated with spillover because suppressing or faking emotions does not resolve underlying affective states, allowing emotional dissonance to persist after work interactions conclude (24, 34). Empirical evidence indicates that such unresolved regulatory strain predicts emotional exhaustion, reduced well-being, and negative mental health outcomes beyond the workplace (33, 36). Spillover is therefore understood as a cumulative resource-based process in which repeated emotional labor constrains employees’ capacity for emotional recovery during non-work time (23, 48).

#### 5.2.3 Role exit

Within emotional labor theory, role exit difficulties are conceptualized as the persistence of emotion regulation processes after formal work interactions have ended, reflecting incomplete disengagement from organizational display rules (22, 23). Employees who rely heavily on surface acting or deep acting to meet emotional display expectations may continue monitoring and managing their emotional expressions beyond the work role, prolonging regulatory effort (23, 47). This sustained regulation maintains emotional dissonance, particularly when expressed emotions diverge from felt emotions, which prevents psychological detachment from work demands (24, 36). As a result, difficulty exiting emotional labor roles contributes to emotional exhaustion by extending resource depletion past the interactional context in which regulation was required (33, 34).

#### 5.2.4 Transition difficulty

Finally, transition difficulties arise when employees must rapidly shift between regulated and unregulated emotional states without sufficient recovery opportunities (23). Frequent initiation and termination of surface or deep acting increases cognitive and physiological activation, particularly in roles characterized by high emotional demands and interpersonal exposure (33, 34). Surface acting is especially detrimental to recovery because it requires ongoing suppression of emotional expression while leaving internal arousal intact, prolonging stress responses after work episodes end (24, 47). Over time, impaired recovery from repeated emotional labor transitions contributes to chronic emotional exhaustion and burnout, especially in the absence of supportive climates that buffer emotion rule dissonance (23, 36).

### 5.3 Differentiations: emotional labor theory vs. sex work personas

Research in occupational psychology shows that workers in high-demand interpersonal professions such as therapists, first responders, medical personnel, and performers frequently engage in controlled dissociative distancing to maintain functioning under stress (22, 32). This boundary work allows individuals to suppress or bracket personal emotions in order to remain composed, effective, and safe in their roles. Sex workers similarly report developing a distinct “work persona,” using controlled dissociation as an adaptive strategy (20, 21, 49). Within these contexts, compartmentalization serves as a cognitive and emotional shield that enhances performance while protecting the core identity from overstimulation or harm.

Although emotional labor theory conceptualizes role enactment as the regulation of emotion and expression within externally defined occupational roles (22, 23, 32), ethnographic research on sex work documents a more identity-explicit process of persona construction (16–21). Rather than merely adjusting emotional displays within a shared professional role, many sex workers describe intentionally creating and inhabiting a distinct work persona that is experienced as separate from their everyday self and designed to manage not only emotional demands, but also safety, stigma, and identity exposure (16–21). While both processes involve emotion regulation, work personas function as bounded intermediary identity states, whereas emotional labor role enactment typically presumes continuity of sense of self across work and non-work contexts (16–23, 32).

## 6 Performance psychology

Because sex workers themselves frequently describe their work personas as forms of acting or performance, understanding how these intentional and sustained roles are psychologically maintained directs attention to research on acting and performance psychology (16, 20–22, 32). Performance psychology and actor-training literatures conceptualize acting as an identity-engaging process rather than a form of emotion regulation, emphasizing the temporary reorganization of self–role boundaries during character embodiment (25, 37, 38). In this framework, acting involves sustained adoption of a character’s perspective, affective stance, and behavioral patterns, which may partially displace ordinary self-referential processing while the role is active (25). Recent neuroscience-informed empirical research suggested that acting can involve partial dampening of autobiographical self-processing, lending mechanistic support to phenomenological accounts of “becoming” the character (25). Within performance psychology, such shifts are considered functional for enactment but heighten the importance of structured identity transition practices following immersion (50). Unlike emotional labor, which presumes continuity of the self across roles, immersive acting explicitly tolerates or encourages dampening of the everyday self in service of character authenticity (37). As a result, the primary psychological risks identified in acting research concern identity boundaries rather than regulatory emotional exhaustion (38).

### 6.1 Performance psychology—psychological strains

Within performance psychology, psychological strain associated with acting is not primarily attributed to emotional intensity itself, but to difficulties managing the relationship between the actor and the enacted role (37, 38). The literature consistently identifies interrelated sources of strain: insufficient disengagement from character, over-identification in which emotional material is experienced as belonging to the personal self rather than the role, and insufficient role absorption that leaves affect inadequately contained during enactment (25, 37, 51). Together, these mechanisms describe poorly regulated role boundary consequences, which is viewed in performance psychology as normative, expected, and reversible, not a signal of individual pathology (38, 50, 51).

#### 6.1.1 Performance psychology—role absorption

Role absorption describes the degree to which an actor becomes experientially embedded in the character’s perspective, emotional stance, and behavioral logic during performance, allowing affect to be organized and expressed through the role rather than the personal self (25). Insufficient role absorption is characterized by persistent self-consciousness, effortful emotion generation, and ongoing monitoring of one’s own reactions, which actors report as destabilizing and emotionally taxing (37, 38). Neuroscience-informed findings indicate that effective acting is associated with reduced engagement of self-processing systems, whereas insufficient absorption may prevent this shift, leaving emotional material inadequately contained and increasing the likelihood of boundary blurring and post-performance distress (25, 51).

#### 6.1.2 Performance psychology—over identification

In acting and performance psychology, over-identification refers to situations in which emotional material is experienced primarily as belonging to the actor’s personal self rather than being mediated through the character (37, 52). When over-identification occurs, actors report heightened vulnerability, increased emotional exposure, and greater difficulty containing intense affect, as emotions are felt as autobiographical rather than role-based (37). This tension highlights the paradox that effective performance often requires partial reduction of self-focus, while insufficient distancing may amplify vulnerability during emotionally demanding roles (25, 37).

#### 6.1.3 Performance psychology—insufficient disengagement

Performance psychology literature consistently identifies insufficient disengagement from character as a primary source of psychological strain for actors, particularly following emotionally intense or immersive performances (37, 38). When actors do not adequately disengage, affective, cognitive, and interpersonal patterns associated with the role may persist beyond performance contexts, contributing to distress, fatigue, and difficulty resuming ordinary social functioning (37). Across this literature, adverse outcomes are attributed to failures of role containment and boundary restoration rather than to acting itself, positioning de-rolling as a preventive identity-regulation process rather than a therapeutic intervention (37, 38).

Crucially, performance psychology literature frames these adverse effects as predictable and normative outcomes of ineffective role boundaries, not as a personal response to trauma, nor as psychological pathology. Acting pedagogy frames these outcomes as failures of role containment, emphasizing that effective deep acting requires the construction of a sufficiently distinct role state, adequate embodiment of the character in order to both produce and absorb emotional intensity without collapsing or spilling into the actor’s autobiographical self, and ability to de-role and return to the self-identity state (52, 53).

## 7 Synthesis and theory: persona-mediated dissociation

Across trauma, emotional labor, and performance psychology, a convergent premise emerges: psychological distancing can function as a context-bound, adaptive regulatory strategy, enabling individuals to tolerate emotionally demanding interactions while preserving overall functioning, with risk arising when distancing becomes rigid, involuntary, or persists beyond its adaptive context (2, 3, 5, 24, 25). Dissociation research conceptualizes such distancing along a continuum from adaptive regulation to maladaptive or clinically impairing dissociation, distinguished by voluntariness, contextual specificity, reversibility, and functional impact (1, 2, 5). Emotional labor theory demonstrates that workers routinely regulate affect and expression to meet occupational demands, with adverse outcomes linked primarily to self-regulatory depletion, emotional dissonance, and impaired recovery rather than identity disruption (22–24, 33, 36). In contrast, performance psychology highlights identity-level role enactment processes, showing that immersive roles can temporarily reorganize self–role boundaries, with strain emerging when disengagement or boundary restoration is insufficient (25, 37, 38).

Ethnographic and qualitative research on sex work intersects these literatures, consistently documenting intentional construction of work personas used to regulate emotion, manage stigma and safety, and create psychological distance described in dissociative terms (16–21). However, no existing framework adequately explains how these practices simultaneously resemble adaptive dissociation, extend beyond emotional labor, and remain distinct from theatrical acting, indicating the need for a model that explicitly centers the work persona as a mediating structure between self, role, and dissociative regulation (2, 5).

### 7.1 Proposed conceptual framework: persona-mediated dissociation

It is at this point of conceptual precipice that direct empirical integration remains limited and existing frameworks reach the boundaries of their explanatory scope. Here, I draw on my subjective lived experience and community-based observation as well as documented sex work scholarship, interdisciplinary occupational theory, trauma-informed dissociation research, and established clinical frameworks to propose a new conceptual framework: *persona-mediated dissociation.*

Persona-mediated dissociation is a proposed as a continuum of patterns characterized by context-specific, identity-mediated psychological distancing in which a deliberately constructed work persona functions as an intermediary structure between the individual’s private self and the emotional, interpersonal, and embodied demands of occupational interaction (16, 18, 20, 21). Rather than involving a diffuse disruption of integration, persona-mediated dissociation is organized through a bounded identity state, such that the persona engages the interaction, while the private self-experiences partial psychological distancing or altered self-experience consistent with dissociative experiences (2, 3). Within this framework, the work persona extends beyond emotional expression regulation, yet cannot be reduced to character performance alone; instead the persona actively structures how experience is appraised, enacted, and encoded, shaping the distribution of affective load and self-referential processing during occupational performance (22, 23, 25).

#### 7.1.1 Model overview

The proposed model conceptualizes persona-mediated dissociation as a state-dependent process in which occupational context activates a work persona that mediates affective load and self-referential processing, producing dissociative-like experiences while the persona is active. Voluntariness, reversibility, and role-exit capacity function as central regulatory mechanisms, while persona use may be adaptive or contribute to spillover and distress (15, 18, 22, 23).

#### 7.1.2 Caveators to the proposed theoretical framework

The proposed persona-mediated dissociation model is not advanced as a universal description of sex workers’ experiences, but as an interpretive continuum based framework that integrates the converging interdisciplinary evidence suggesting that some sex workers employ identity-mediated, dissociative-illustrative regulatory strategies in specific occupational contexts.

As the term *mediated* suggests, not all manifestations of this proposed phenomenon are purely adaptive and beneficial, nor maladaptive and psychologically concerning. Persona-mediated dissociation is not defined by the absence of psychological cost, nor do I propose persona-mediated dissociation as a diagnostic category, but as an analytic and interdisciplinary framework for understanding how role-based identity persona regulation intersects with dissociative processes in occupational, specifically sex work contexts.

Persona-mediated dissociation is therefore conceptualized as neither inherently adaptive nor pathological, but as a regulatory configuration whose effects depend on how the persona is entered, sustained, and exited. When persona use is intentional, context-bound, and reversible, it may resemble or function as adaptive dissociation by modulating affective intensity, reducing self-referential exposure, and preserving task engagement under conditions of heightened interpersonal demand (2, 5, 26). Conversely, and calling upon the psychological strains that accompany emotional labor theory and performance psychology, when persona boundaries become rigid, poorly disengaged, or chronically activated beyond the work context, the same processes may contribute to spillover, emotional numbing, identity transition difficulty, or overall distress (6, 37, 38, 51).

By explicitly centering the work persona as a mediating structure, persona-mediated dissociation offers a framework capable of integrating sex workers’ lived descriptions of adaptive, maladaptive, or clinical dissociative features as well as the creation of intentional personas at work, avoiding the stigmatizing trauma-essential assumptions that have historically dominated sex work literature. This model accounts for why dissociative features in sex work may be experienced as skilled, intentional, and protective in some contexts, yet distressing or impairing in others, depending on contextual demands, structural constraints, and the integrity of role boundaries (5, 18, 20, 21).

##### 7.1.2.1 Differentiation from adjacent constructs

Persona-mediated dissociation is not reducible to emotional labor, which centers emotion regulation within a continuous self and predicts strain via self-regulatory depletion rather than identity-state mediation (22, 23). It is distinct from performance or theatrical acting, which involves immersive role embodiment without necessarily entailing dissociative phenomenology, and locates risk in over-identification or insufficient de-rolling rather than dissociative regulation (25, 37). Finally, it is distinct from clinical dissociation, defined by involuntariness, cross-context pervasiveness, impaired reversibility, and clinically significant distress or impairment (1, 27). Incremental validity is therefore expected insofar as persona-mediated processes predict occupational spillover and distress specifically under conditions of impaired role exit or structural constraint, rather than as a function of trauma exposure alone (5, 15).

### 7.2 Testable hypotheses

*H*1 (Context specificity). Dissociative experiences are expected to be more pronounced during work-related interactions than in non-work contexts among individuals who report activation of a work persona.

*H*2 (Voluntariness and reversibility). The voluntariness of persona entry and the reversibility of persona exit are expected to moderate the association between dissociative phenomenology and psychological distress, such that dissociative experiences will be weakly associated with distress when voluntariness and reversibility are high, but more strongly and positively associated with distress when they are low.

*H*3 (Role exit mediation). Difficulty disengaging from the work persona following an interaction is expected to mediate the relationship between frequency of persona activation and post-work outcomes, including emotional numbing, spillover into non-work domains, and impaired psychological recovery.

*H*4 (Incremental validity). Indicators of persona-mediated dissociation are expected to explain unique persona-linked variance in emotional spillover, emotional numbing, and identity strain beyond established emotional labor constructs, including surface acting, deep acting, and emotional dissonance.

*H*5 (Threshold effects). The association between persona activation and psychological distress is expected to be nonlinear, such that low-to-moderate, context-bound persona use will be neutral or adaptive, whereas high-frequency or prolonged activation, or impaired persona exit, will predict increased distress and functional impairment over time.

## 8 Discussion

Sex work has long occupied a contested position within academic, political, and clinical discourse, often functioning less as a singular occupational category and more as a site onto which broader debates about gender, morality, consent, and power are projected (17, 20, 21, 39). Combined with methodological restraints, scholarly research tends to examine sex work through polarizing lenses—exploitation versus agency, victimization versus choice, and harm versus empowerment (17, 20, 21). Each of these perspectives has generated important insights, yet both extremes struggle to accommodate variability in lived experiences (39).

Global estimates suggest that approximately 40–42 million people engage in sex work (7). Over 80% of empirical samples consist of women, reflecting a strong research concentration on cisgender female sex workers, particularly those in visible or criminalized contexts (7). Existing mental health research on sex work remains heterogeneous, limiting consistent operational definitions across studies. The frequent conflation of consensual sex work with human trafficking further complicates interpretation of available data and necessitates careful contextual differentiation (39).

Accordingly, the present article and framework is proposed within the constraints of the existing literature. Further empirical research across cultures, genders, venues, legal contexts, intersecting identities, and individual differences is required before findings can be generalized.

This paper advances persona-mediated dissociation as a testable, context-sensitive framework for understanding how some sex workers manage intense interpersonal, emotional, and embodied occupational demands through intentional identity mediation, rather than through global psychological fragmentation or assumed psychopathology (2, 5, 20, 21). By integrating contemporary dissociation theory, emotional labor research, performance psychology, and sex work scholarship, the framework situates dissociative-like experiences within occupational role regulation, emphasizing function, context, and boundary integrity as central explanatory dimensions (5, 22, 25, 37).

### 8.1 Work modality, duration, and psychological outcomes

Work context may also shape sex workers’ dissociative strategies. Indoor workers, outdoor workers, lower contact workers, emotionally focused, and online creators face different role demands, identity risks, and stigma (40, 41).

#### 8.1.1 Online work persona

Online workers, in particular, contend with identity traceability and surveillance, often eliminating personal social media to avoid cross-linking (54).

Nelson (19) defines context collapse for sex workers as the breakdown of audience boundaries on social media, where content intended for clients can be simultaneously accessed by family members, employers, or hostile publics, increasing the risk of stigma, surveillance, and harm. In response, many sex workers intentionally avoid maintaining personal social media accounts and instead operate exclusively through carefully managed work personas to prevent unintended audience overlap and identity exposure (19). While this strategy enhances privacy and safety, Nelson (19) notes that it also constrains core self-expression and can produce a sense of invisibility or fragmentation, as workers must continuously manage and limit how their identities can exist online.

#### 8.1.2 Extended engagement work persona

Extended engagements such as travel-based arrangements, long-duration sugar relationships, or “gold digging” introduce distinct psychological conditions for sex workers by requiring sustained performance of the work persona across extended, minimally interrupted periods rather than discrete, time-limited encounters (20, 21, 42). Compared to short interactions, longer engagements reduce the frequency of repeated role entry and exit, a pattern that emotional-labor research associates with greater short-term performance stability but also increased demands on sustained affect regulation (22, 32).

Qualitative studies of escorting and sugar dating indicate that prolonged intimacy and shared daily routines intensify emotional labor, as workers must continuously manage presentation, availability, and boundaries without consistent access to backstage spaces typically used for emotional decompression (20, 21, 42, 43). Other workers describe extended arrangements as subjectively easier in the short term because the work persona becomes more routinized, reducing the need for constant negotiation or rapid identity shifting characteristic of brief encounters (20, 21, 49).

Research on immersive role performance and emotional labor suggests that sustained role embodiment without structured disengagement is associated with increased emotional carryover and difficulty returning to baseline functioning, particularly when rest, privacy, or role separation are limited (32).

### 8.2 Limitations

The conclusions drawn in this perspective are constrained by limitations in both the existing literature on dissociation in sex workers and the conceptual nature of the proposed framework (1, 11–15, 31). The present paper is conceptual and integrative in nature, synthesizing dissociation theory, emotional labor, performance psychology, and sex work scholarship, while also being informed by experiential knowledge, rather than reporting original empirical findings; accordingly, the proposed framework of persona-mediated dissociation should be understood as a theory-building heuristic rather than a validated explanatory model or diagnostic framework (1–3).

Interpretation is further constrained by the limited, dated, and methodologically varied empirical literature on dissociation in sex workers (11–15, 31). Additionally, no validated instruments currently exist to directly assess occupational personas, identity permeability, or voluntary versus involuntary dissociative transitions in non-clinical populations, requiring reliance on proxy constructs drawn from adjacent literatures (1–3). The framework also draws heavily on qualitative and ethnographic accounts of sex work, which provide rich phenomenological insight but do not establish prevalence, causality, or boundary conditions across diverse work contexts (14, 16–21).

### 8.3 Directions for future research

At present, it remains unknown whether *persona-mediated dissociation*, as described in this paper, represents a distinct and empirically observable phenomenon, nor if it is common or rare among sex workers. No empirical studies have systematically examined its existence, prevalence, frequency, intensity, or functional variability across sex work contexts (11–13, 15, 20, 21, 31). Nor is there empirical evidence clarifying whether identity-mediated dissociative processes are predominantly protective, harmful, neutral, or dynamically contingent on specific occupational, interpersonal, or structural factors (2, 6, 13, 15).

A critical priority for future research is the development and validation of measurement approaches capable of distinguishing intentional, context-bound dissociation from involuntary or clinically significant dissociative processes. Existing dissociation measures largely presume trauma-based pathology and do not assess voluntariness, reversibility, role entry, or role exit capacity in occupational settings (1–3). Adapting or extending dissociation instruments to include occupational context, identity mediation, and boundary integrity would enable empirical testing of whether persona-mediated processes predict outcomes above and beyond established emotional labor constructs (5, 23, 24).

Critically, the field also lacks data addressing whether persona-mediated dissociation confers short-term regulatory benefits while carrying potential long-term costs, whether it becomes maladaptive only beyond certain thresholds of intensity, frequency, or chronicity, or whether specific occupational, interpersonal, or structural conditions increase the likelihood that identity-mediated distancing shifts from adaptive regulation to distressing or clinically impairing dissociation (2, 5).

Currently, no clinical framework exists to distinguish intentional, context-specific dissociation from involuntary dissociation when both present with features such as identity fragmentation, physiological blunting, or a buffered sense of self. There is also no consensus regarding thresholds at which occupational dissociation becomes clinically concerning, particularly when executive functioning and agency remain intact. Longitudinal and experience-sampling designs are particularly well suited to examining these temporal dynamics and threshold effects, allowing researchers to distinguish state-dependent regulatory responses from persistent or cumulative patterns (29).

Future research should explicitly test whether persona-mediated processes predict outcomes above and beyond traditional emotional labor measures, and whether voluntariness, reversibility, and role exit capacity moderate associations between dissociative phenomenology and distress (2, 5, 23). Mixed-method designs that combine qualitative mapping of persona boundaries with longitudinal or experience-sampling approaches are particularly well suited to evaluating state-dependent versus persistent effects (11, 29). Critically, such work should incorporate structural determinants such as criminalization, stigma, surveillance, and access to peer support, as central variables rather than background context, in keeping with contemporary sex work research priorities (15, 18) (Rössler, 2018).

Finally, there is little guidance for researchers or clinicians on how to determine whether work personas function as protective regulatory strategies or contribute to distress, nor on therapeutic stance, including whether persona use should be supported, modified, or discouraged, or how to facilitate safe role exit, decompression, and post-work integration among individuals who rely on personas for emotional labor (2, 15, 20–24).

### 8.4 Clinical and ethical implications

In the absence of such evidence, it is not possible to determine whether persona construction should be supported, discouraged, or neutrally explored within clinical settings, nor whether psychoeducation around persona use would be protective, unnecessary, or potentially harmful.

As a result, clinicians currently lack an empirical basis from which to make informed judgments about whether to normalize, pathologize, reinforce, or intervene upon sex workers’ use of work personas or identity-based distancing strategies. Without clarity regarding prevalence, function, and risk conditions, clinical responses risk being driven by theoretical assumptions, moral frameworks, or trauma-essentialist interpretations rather than evidence-informed, context-responsive assessment. This uncertainty underscores the need for empirical research capable of distinguishing between adaptive, maladaptive, and clinically significant forms of identity-mediated dissociation in sex work contexts before prescriptive clinical guidance can be responsibly offered.

Exposure to this interdisciplinary synthesis may equip clinicians with a broader conceptual lens through which to understand sex workers’ descriptions of identity shifts, emotional distancing, or altered self-experience during work, drawing on emotional labor theory, performance psychology, and contemporary dissociation research rather than relying solely on trauma-pathology frameworks. This perspective encourages clinicians to attend not only to the presence of dissociative-like experiences, but to their functional role, contextual specificity, voluntariness, reversibility, and associated distress or impairment, which are central distinguishing features across dissociation models (1–3).

Viewed through this lens, clinical assessment may be oriented toward evaluating outcomes rather than assuming dysfunction: whether identity-mediated distancing supports occupational functioning, emotional containment, and boundary maintenance, or whether it contributes to spillover, distress, impaired recovery, or loss of control. By foregrounding function and impact over mere existence, clinicians may be better positioned to avoid premature pathologization while remaining attentive to indicators of maladaptive or clinically significant dissociation. Importantly, this framework does not prescribe endorsement or discouragement of persona use, but underscores the need for individualized, context-responsive clinical understanding in the absence of definitive empirical guidance.

By articulating persona-mediated dissociation as a testable, context-sensitive framework grounded in interdisciplinary theory and sex workers’ lived realities, this paper aims to advance empirical inquiry and public health understanding of occupational mental health in sex work beyond pathology-based models toward structurally informed, evidence-responsive research.

## Data Availability

The original contributions presented in the study are included in the article/supplementary material, further inquiries can be directed to the corresponding author.
